# Prognostic Value of Microscopic Tumor Necrosis in Adrenal Cortical Carcinoma

**DOI:** 10.1007/s12022-023-09760-6

**Published:** 2023-03-23

**Authors:** Michaela Luconi, Giulia Cantini, Rachel S. van Leeuwaarde, Rogina Roebaar, Laura Fei, Arianna Pia Propato, Raffaella Santi, Tonino Ercolino, Massimo Mannelli, Letizia Canu, Ronald R. de Krijger, Gabriella Nesi

**Affiliations:** 1 Endocrinology Unit, Department of Experimental and Clinical Biomedical Sciences “Mario Serio”, University of Florence, Viale Gaetano Pieraccini 6, Florence, 50139 Italy; 2 Centro di Ricerca E Innovazione Sulle Patologie Surrenaliche, AOU Careggi, 50134 Florence, Italy; 3 ENS@T Center of Excellence, Florence, Italy; 4 Department of Endocrine Oncology, University Medical Center Utrecht, Utrecht, 3584 CX The Netherlands; 5 Pathology Section, Department of Health Sciences, University of Florence, Viale Gaetano Pieraccini 6, Florence, 50139 Italy; 6 Endocrinology Unit, Careggi University Hospital (AOUC), Florence, 50139 Italy; 7 Princess Maxima Center for Pediatric Oncology, Utrecht, 3584 CS The Netherlands; 8 Department of Pathology, University Medical Center Utrecht, Utrecht, 3584 CX The Netherlands

**Keywords:** Weiss score, ACC, Survival analysis, Principal component analysis

## Abstract

Adrenal cortical carcinoma (ACC) is an uncommon neoplasm with variable prognosis. Several histologic criteria have been identified as predictors of malignancy in adrenal cortical tumors. The Weiss score is the system most widely employed for diagnostic purposes, but also possesses prognostic value. We aim to determine the relative impact of each Weiss parameter on ACC patient survival. A multicenter retrospective analysis was conducted on a series of 79 conventional ACCs surgically treated at the Florence and Utrecht centers of the European Network for the Study of Adrenal Tumors (ENSAT). Weiss classification was recapitulated using principal component analysis (PCA). The Kaplan–Meier and Cox multivariate regression analyses were applied in order to estimate the prognostic power of Weiss versus other clinical parameters. PCA reduced the nine Weiss parameters to the best fitting 4-component model, each parameter clustering with a single component. Necrosis and venous invasion clustered together with the highest scores, thus establishing the most relevant component (Component 1) to explain Weiss distribution variability. Only Component 1 significantly predicted overall survival (OS, log-rank = 0.008) and disease-free survival (DFS, log-rank < 0.001). When considering the prognostic power of Weiss parameters, necrosis alone could independently assess OS (log-rank = 0.004) and DFS (log-rank < 0.001) at both the Kaplan–Meier and multivariate Cox regression analyses [hazard ratio (HR) = 7.8, 95% confidence interval [CI] = 1.0–63.5, *p* = 0.05, and HR = 12.2, 95% CI = 1.6–95.0, *p* = 0.017, respectively]. The presence of necrosis significantly shortened time to survival (TtS) and time to recurrence (TtR), 57.5 [31.5–103.5] vs 34 [12–78] months (*p* = 0.05) and 57.5 [31.5–103.5] vs 7 [1.0–31.5] months (*p* < 0.001), respectively. Our study suggests that, of the Weiss parameters, necrosis is the most powerful adverse factor and the best predictor of OS and DFS in ACC patients.

## Introduction

Adrenal cortical carcinoma (ACC) is a rare and often aggressive malignancy, with a 5-year survival less than 20% in patients with metastatic disease [1]. In addition to conventional clinical factors such as tumor size, stage, functional activity, total Weiss score, and Ki67 proliferation index, a number of molecular markers have recently been recognized to improve accuracy for survival prediction [2–5], particularly in metastatic cases [6, 7]. However, implementation of validated histologic parameters may also be of use in identifying patients for correct stratification and individualized follow-up.

The Weiss score is the most widely applied histologic multiparameter scoring system for the classification of adrenal cortical tumors in adults. It is based on 9 histologic criteria, with the inherent problems of subjective scoring and intra- and interobserver variability. While endorsed in the most recent WHO blue book on endocrine and neuroendocrine tumors, the fact that other, newer, systems have been developed, such as the Helsinki score and reticulin algorithm, indicates room for improvement [8–10]. Importantly, not all 9 parameters may carry the same diagnostic and prognostic relevance, but so far no studies have addressed this issue [5].

Principal component analysis (PCA) is a statistical tool applicable to large datasets for reducing the number of correlated variables, increasing interpretability, but at the same time minimizing information loss [11]. This is achieved by a linear transformation of the initial dataset into new uncorrelated variables that in turn maximize variance. The new variables, named “principal components,” are not generated a priori, rendering PCA an adaptive data analysis technique.

The current study aimed to evaluate the prognostic value of individual Weiss parameters in a large ACC dataset, in an attempt to simplify and prioritize their analysis adopting a PCA approach.

## Materials and Methods

### Patients and Ethical Approval

We retrospectively analyzed a series of 79 conventional ACCs following surgical removal of the tumor at Careggi University Hospital, Florence, Italy (*n* = 57) or University Medical Center Utrecht, Utrecht, The Netherlands (*n* = 22) between 1999 and 2021. Formalin-fixed paraffin-embedded tumor samples were available for immunohistochemical analysis. The study design was reviewed and approved by the Careggi University Hospital Ethical Committee (Prot. 2011/0020149 - Rif CEAVC Em. 2019–201 26/11/2019). The Medical Ethical Committee (MERC) of the University Medical Center Utrecht confirmed that the Medical Research Involving Human Subjects Act (WMO) did not apply to this study and no official approval was required under WMO. The patients recruited in Florence gave their written informed consent, which was not necessary for the Dutch cohort.

### Pathologic Analysis of ACC Samples

Histologic diagnosis of ACC was carried out by two independent reference pathologists on tumor tissue removed at surgery. Tumor specimens were evaluated according to the Weiss scoring system, in which the presence of three or more criteria highly correlates with malignant behavior [12]. The Ki67 labeling index (LI) was estimated on digitalized glass slides, after immunohistochemical staining with anti-human Ki67 antibody (1:40 dilution, MIB-1, Dako, Carpinteria, CA, USA). Areas with the highest labeling were manually identified, Ki67-positive nuclei were counted in 1000 tumor cells, and Ki67 LI was expressed as the percentage of labeled cells, using the Ki67 algorithm available in the Picture Archiving and Communication System (PACS) (Sectra Medical, Linkoping, Sweden). Tumor stage was assessed according to the revised 8th edition of the TNM classification of ACC proposed by ENSAT [13].

### Statistical Analysis

Continuous variables with normal distribution were presented as mean (standard deviation [SD]) and non-normal variables as median (interquartile range [IQR]). Categorical variables were expressed as counts and percentages. Statistical analysis was performed with SPSS 26.0 (Statistical Package for the Social Sciences, Chicago, IL, USA) for Windows. A *p*-value less than 0.05 was considered statistically significant. Possible associations were investigated using the *χ*^2^ test for categorical variables and Pearson’s correlation for continuous variables. Comparison between two groups of data was accomplished using Student’s *t*-test for normally distributed variables and *U* Mann–Whitney test for non-parametric variables. Overall survival (OS) and disease-free survival (DFS) are defined as the probability (ranging from 0 to 1) that a patient diagnosed with the disease is still alive (OS) or free from the disease (DFS) at a time point from surgery. Survival analysis was estimated through the Kaplan–Meier method, and differences between groups were calculated by the log-rank test. Only variables significantly predicting OS and DFS at the Kaplan–Meier analysis were used for univariate and multivariate Cox regression analyses to define hazard ratio (HR) and 95% confidence intervals (CI). PCA was performed on the ACC cases to ascertain the best model for clustering and reducing the number of Weiss parameters (i.e., diffuse architecture, confluent necrosis, clear cells comprising less than 25% of the tumor, venous invasion, sinusoidal invasion, capsular invasion, high nuclear grade, mitotic count of > 5 mitoses per 50 high-power fields (10 mm^2^), and atypical mitotic figures) in an unsupervised and un-hierarchical manner.

## Results

**Fig. 1:**
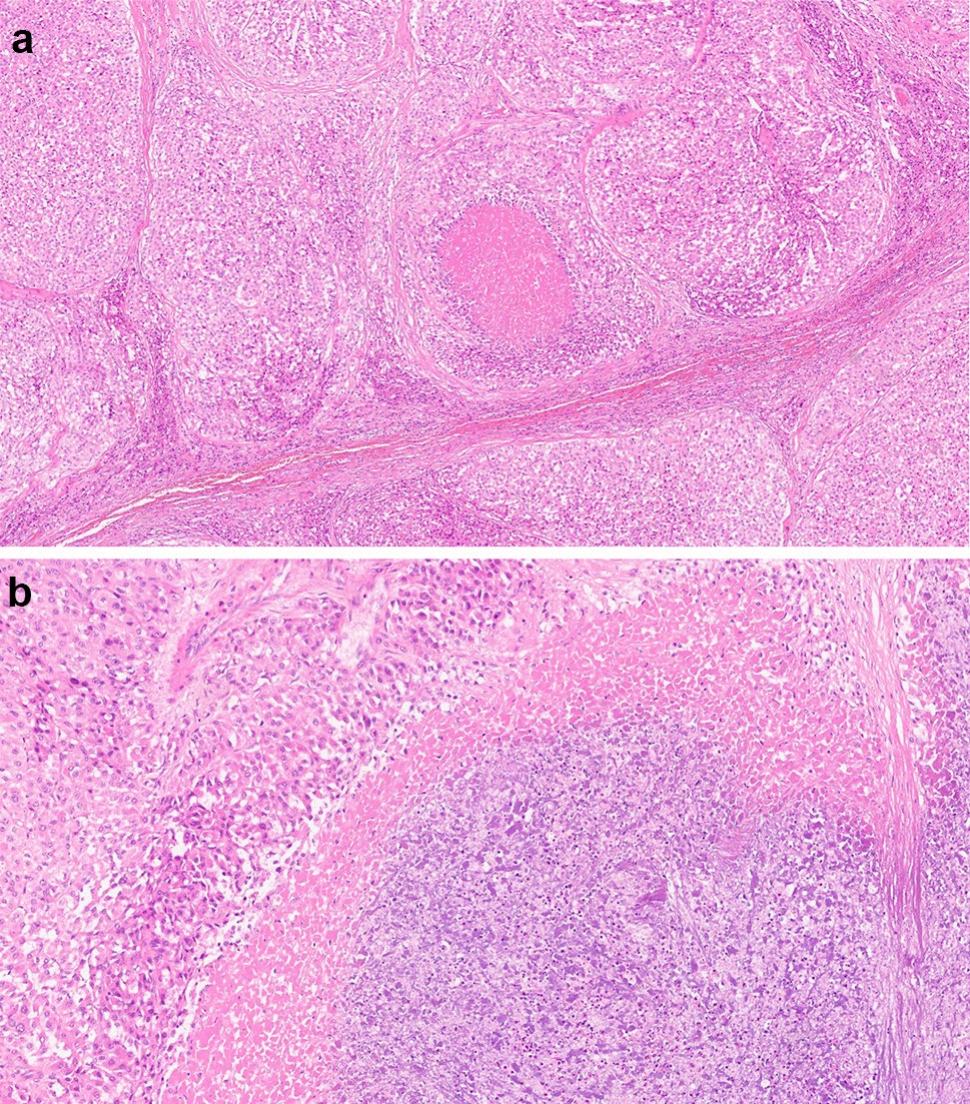
Coagulative necrosis. Representative section of high-grade ACC with comedo-type necrosis (**A**, 40 ×). High-power view of abrupt transition from viable to non-viable tumor without evidence of intervening tissue (**B**, 200 ×)

The main clinico-pathologic characteristics of ACC patients (*n* = 79) are illustrated in Table 1. Our cohort comprised 52 (64%) female and 27 (34%) male patients, with a mean ± SD age of 49.2 ± 15.1 years. Increased hormonal secretion was observed in the majority of patients (*n* = 47/75; 59%) with glucocorticoid excess being the most frequent aberration (*n* = 32/47; 68%). There were 28 (35%) non-secreting tumors, and for 4 patients relevant data were not available. Tumor stage was I in 12 (15%) patients, II in 30 (38%), III in 20 (25%), and IV in 17 (22%). Mean ± SD tumor size was 11.2 ± 5.9 cm. Among the 69 patients for whom resection margin status (RMS) was known, 44 (56%) had undergone complete surgical resection (R0). Median [IQR] Weiss score was 6 [5–7]; distribution of each Weiss parameter in our cohort is detailed in Table 2. In particular, necrosis was a frequent finding, observed in 61 (77%) patients (Fig. 1). Mean ± SD Ki67 LI was 27.3 ± 25.7. Median [IQR] follow-up was 39 [16–82] months and time to recurrence (TtR) 11 [3–59] months. Overall, 29 (37%) patients died of the disease and 42 (53%) experienced recurrence.

**Table 1:** ACC patient characteristics. Data from the ACC cohort (*n* = 79) are expressed as mean ± SD, for continuous parametric and median [interquartile] for non-parametric variables and as absolute number and percentage of patients for non-continuous variables

Variable	Values
Age (years)	49.2 ± 15.1 (range 10–79)
Sex	F: 52 (64%)
	M: 27 (34%)
Secretion	NS: 28 (35%)
	GC: 32 (41%)
	A: 14 (18%)
	MC: 1 (1%)
	NA: 4 (5%)
Tumor size (cm)	11.2 ± 5.9 (range 2–30)
Stage	I: 12 (15%)
	II: 30 (38%)
	III: 20 (25%)
	IV: 17 (22%)
Total Weiss score	6.0 [5–7] (range 3–9)
Ki67 LI	27.3 ± 25.7 (range 2–96)
RMS	R0: 44 (56%)
	R1: 25 (32%)
	NA: 10 (12%)
Recurrence	Y: 42 (47%)
	N: 37 (53%)
Death	Y: 29 (37%)
	N: 50 (63%)
FU (months)	39 [16–82] (range 1–258)
TtR (months)	11 [3–59–82] (range 0–258)

*FU* follow-up time, *TtR* time to recurrence, *NS* non-secreting, *GC* glucocorticoids, *A* androgens, *MC* mineralocorticoids, *NA* not available, *LI* labeling index, *RMS* resection margin status, *Y* yes, *N* no

**Table 2:** Weiss parameter distribution in the ACC cohort. Number and percentage of positive or negative cases for each Weiss parameter are indicated in the total cohort and R0 patients

	Total cohort (*n* = 79)		R0 patients (*n* = 44)	
Weiss parameter	**Negative** ***n* (%)**	**Positive** ***n* (%)**	**Negative** ***n* (%)**	**Positive** ***n* (%)**
High nuclear grade	19 (24%)	60 (76%)	13 (30%)	31 (70%)
Atypical mitosis	42 (53%)	37 (47%)	28 (64%)	16(36%)
> 5 mitoses per 50 high-power fields (10 mm^2^)	13 (16%)	66 (84%)	9 (20%)	35 (80%)
Clear cells ≤ 25%	11 (14%)	68 (86%)	4 (9%)	40 (91%)
Diffuse architecture	22 (28%)	57 (72%)	8 (18%)	36 (82%)
Necrosis	18 (23%)	61 (77%)	13 (30%)	31 (70%)
Venous invasion	40 (51%)	39 (49%)	24 (54%)	20 (46%)
Sinusoidal invasion	38 (48%)	41 (52%)	21 (48%)	23 (52%)
Capsular invasion	35 (44%)	44 (56%)	18 (41%)	26 (59%)

**Fig. 2:**
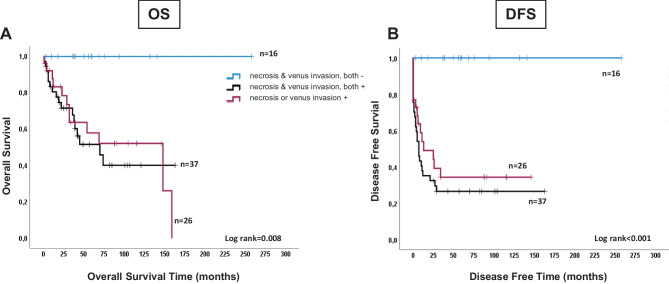
Overall (OS) and disease-free (DFS) survival predictive value of Component 1. The Kaplan–Meier analysis of OS (**A**) and DFS (**B**) in ACC patients stratified into three classes according to Component 1. Statistically significant differences in the Kaplan–Meier analysis were evaluated as log-rank; number of patients is indicated

PCA of the nine Weiss parameters, conducted stepwise from an autovalue > 1, identified a 4-component model with a cumulative variance of 60%. Direct Varimax rotation was then applied to ensure the best fitting clustering and optimization of parameter variance, mostly attributable to a single component for each parameter (Table 3). Component 1, combining necrosis and venous invasion, accounted for almost 25% of the Weiss score variance. To assess the predictive value of the four components, we evaluated OS and DFS by the Kaplan–Meier analysis. When stratifying patients into three classes according to the Weiss parameters clustered in each component, only Component 1 significantly predicted OS (log-rank = 0.008) and DFS (log-rank < 0.001) (Fig. 2).

**Table 3:** PCA analysis of the nine Weiss parameters gave the best fitting model of four components. PCA analysis was performed by SPSS 26.00 on data from the ACC cohort (*n* = 79). Pattern matrix related to a 4-component model, after Varimax rotation and missing pairwise

**Weiss parameter**	**Component 1**	**Component 2**	**Component 3**	**Component 4**
Necrosis	0.801			
Venous invasion	0.744			
Sinusoidal invasion		0.808		
Capsular invasion		0.654		
Diffuse architecture		0.646		
Atypical mitosis			0.879	
** > **5 mitoses per 50 high-power fields (10 mm^2^)			0.657	
Nuclear atypia			0.590	
Clear cells ≤ 25%				0.836

Data are expressed as correlation values between parameters and components > 0.450, and are indicated in descending order. Barlett’s test value for adequacy = 0.548 and significance of the correlation matrix *p* < 0.001. Descending weight of the single components from Component 1 to 4 for covering variance of Weiss score

To determine which parameters best correlated with death and recurrence, we performed regression analysis on continuous variables, i.e., age, tumor stage, tumor size, Weiss score, and Ki67 LI, as well as dichotomized variables, such as presence or absence of necrosis and venous invasion, grouped tumor stage (I–II vs III–IV), tumor size (cut-off = 6 cm), RMS (R0 vs R1), total Weiss score (cut-off value = 6), and Ki67 LI (cut-off value = 20%) (Table 4). Of the Weiss parameters, necrosis alone was significantly associated with death (*r*/*χ*^2^ = 9.7, *p* < 0.002) and recurrence (*r*/*χ*^2^ = 21.2, *p* < 0.001), while venous invasion correlated with recurrence only (*r*/*χ*^2^ = 10.7, *p* = 0.001). In order to test whether RMS could override microscopic features in relation to outcome, we re-ran regression analysis on completely resected tumors (R0). Necrosis remained the only histologic factor related to both relapse and death (Table 4).

**Table 4:** Regression analysis of correlation between death and recurrence with ACC clinico-pathologic parameters

	**Total cohort (*n* = 79)**		**R0 patients (*n* = 44)**	
**Variable**	**Death** ***r*/*χ***^**2**^	**Recurrence** ***r*/*χ***^**2**^	**Death** ***r*/*χ***^**2**^	**Recurrence** ***r*/*χ***^**2**^
Necrosis	9.7	21.2	4.7	10.5
	*0.002*	< *0.001*	*0.029*	*0.001*
Venous invasion	3.0	10.7	2.1	5.5
	0.085	*0.001*	0.152	*0.019*
RMS	11.0	14.5	-	-
(R0/R1)	< *0.001*	< *0.001*		
Age	0.256	0.239	0.261	0.411
	*0.024*	*0.034*	0.086	*0.006*
Tumor size	0.102	0.317	-0.063	0.192
	0.376	*0.004*	0.687	0.211
Tumor stage	0.343	0.580	0.080	0.448
	*0.002*	< *0.001*	0.605	*0.002*
Total Weiss score	0.106	0.273	0.064	0.211
	0.352	*0.015*	0.682	0.170
Ki67 LI	0.157	0.186	0.195	0.008
	0.256	0.179	0.205	0.960
Dichotomized tumor size	5.1	17.7	1.5	9.4
(cut-off 6 cm)	*0.024*	< *0.001*	0.222	*0.002*
Grouped tumor stage	5.6	15.8	0.45	4.3
(I-II vs III-IV)	*0.018*	*0.000*	0.832	*0.038*
Dichotomized Weiss score	1.6	2.6	0.134	0.6
(cut-off 6)	0.200	0.110	0.714	0.447
Dichotomized Ki67 LI	1.3	1.9	0.049	0.052
(cut-off 20%)	0.248	0.172	0.825	0.820

Necrosis, venous invasion, and resection margin status (RMS) are dichotomized variables (presence or absence); age, tumor size, tumor stage, total Weiss score, and Ki67 labeling index (LI) are considered continuous variables; grouped tumor stage and dichotomized Weiss score, tumor size, and Ki67 LI are non-continuous variables; *r*/*χ*^2^ is reported for continuous and categorical variables, respectively; *p* values are indicated (significant in italics)

We therefore focused on necrosis to estimate its predictive power for survival. Necrosis was identified in 61 (77%) of all cases, occurring in 47/61 (77%) of tumors with Weiss score ≥ 6 and in 14/61 (23%) with Weiss score < 6 (*r*/*χ*^2^ = 21.8, *p* < 0.001), with a higher prevalence in masses measuring ≥ 6 cm than in those < 6 cm (92% vs 8%, respectively; *r*/*χ*^2^ = 24.1, *p* < 0.001). Necrosis was present in 27/61 (44%) low stage (I–II) cases and absent in 3/18 (17%) high stage (III–IV) cases (*r*/*χ*^2^ = 6.2, *p* = 0.012). Tumors with necrosis also displayed higher Ki67 LI and a higher risk of death and recurrence than tumors without necrosis; only one death (1/18; 6%) and one recurrence (1/18; 6%) were recorded in cases without microscopic necrosis. In the R0 subgroup, the percentage of tumors without necrosis was higher than in the whole cohort (29.5% and 22.8%, respectively), while necrosis retained its significance with recurrence, death, and TtR (Table 5).

**Table 5:** ACCs with necrosis exhibited aggressive clinico-pathologic features

	Total cohort (*n* = 79)			R0 patients (*n* = 44)		
Variable	**Absence necrosis** **(*n* = 18)**	**Presence** **necrosis (*n* = 61)**	***p* (*χ***^***2***^**)**	**Absence necrosis (*n* = 13)**	**Presence necrosis (*n* = 31)**	***p* (*χ***^***2***^**)**
Age	46.8 ± 16.9	46.9 ± 14.6	0.444	47.9 ± 15.4	49.6 ± 13.2	0.720
Sex (F:M)	11:7	41:20	0.631 (0.230)	7:6	19:12	0.647 (0.210)
Secretion			0.139 (6.9)			0.441 (3.7)
N	7	21		5	6	
GC	4	28		3	10	
A	5	9		3	6	
MC	1	0		1	0	
NA	1	3		0	0	
Tumor size (cm)	7.3 ± 6.5	12.4 ± 5.2	< *0.001*	7.2 ± 7.5	12.0 ± 5.2	*0.020*
Stage			< *0.001* (*23.3*)			< *0.001* (*19.3*)
I	9	3		8	1	
II	6	24		3	16	
III	2	18		2	12	
IV	1	16		0	2	
Total Weiss score	4.2 ± 1.3	6.7 ± 1.4	< *0.001*	4.3 ± 1.4	6.4 ± 1.3	< *0.001*
Ki67 LI	8.6 ± 6.6	31.6 ± 26.5	< *0.001*	11.1 ± 11.2	27.4 ± 23.5	*0.003*
RMS			*0.037* (*4.3*)	-	-	-
R0	13	31				
R1	2	23				
NA	3	8				
Recurrence			< *0.001* (*21.2*)			*0.001* (*10.6*)
N	17	20		13	15	
Y	1	41		*0*	16	
Death			*0.002* (*9.7*)			*0.029* (*4.7*)
N	17	33		13	22	
Y	1	28		0	9	
TtS (months)	57.5 [31.5–103.5]	34 [12–78]	*0.050*	56 [37–95]	42 [22–88]	0.418
TtR (months)	57.5 [31.5–103.5]	7 [1.0–31.5]	< *0.001*	56 [37–95]	25 [4–70]	*0.013*
Dichotomized tumor size (cut-off 6 cm)			< *0.001 (24.1*)			< *0.001 (16.4)*
<	11	5		9	3	
≥	7	56		4	28	
Tumor stage			*0.012* (*6.2*)			0.060 (3.5)
I–II	15	27		11	17	
III–IV	3	34		2	14	
Dichotomized Weiss score (cut-off 6)			< *0.001 (21.8)*			< *0.001 (11.4)*
<	15	14		10	7	
≥	3	47		3	24	

Data are expressed as mean ± SD and frequency for continuous and categorical variables, respectively; TtS and TtR are expressed as median [interquartile]; *p* values are indicated (significant in italics) for Student’s *t*-test analysis for continuous parametric variables, *U* Mann–Whitney test for continuous non-parametric or for *χ*^2^ test (in brackets) for categorical variables

*NS* non-secreting, *GC* glucocorticoids, *A* androgens, *MC* mineralocorticoids, *NA* not available, *LI* labeling index, *RMS* resection margin status, *N* no, *Y* yes, *TtS* time to survival (TtS), *TtR* time to recurrence

**Fig. 3:**
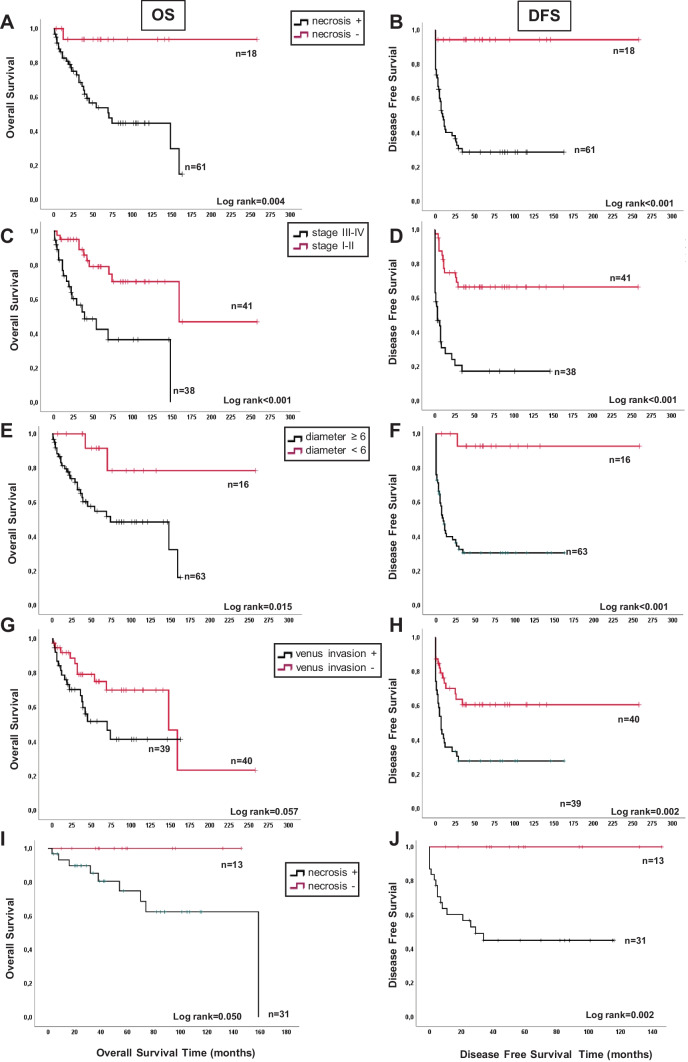
Comparison between survival predictive value of different parameters in the ACC cohort. The Kaplan–Meier analysis of OS and DFS in ACC patients stratified into two classes according to the presence of necrosis (**A**, **B**), tumor stage (**C**, **D**), venous invasion (**E**, **F**) tumor size (**G**, **H**, diameter < or ≥ 6 cm). Necrosis maintained its predictive power for both OS (**I**) and DFS (**J**) in the R0 subgroup. Statistically significant differences in the Kaplan–Meier analysis were evaluated as log-rank; number of patients is indicated

The impact of each Weiss variable on OS and DFS was assessed by the Kaplan–Meier analysis. Necrosis alone proved to significantly predict OS (log-rank = 0.004, Fig. 3A) and DFS (log-rank < 0.001, Fig. 3B), with results comparable to those of dichotomized tumor stage (Fig. 3C and D) and size (Fig. 3E and F). Venous invasion significantly predicted DFS (log-rank = 0.002), but not OS (log-rank = 0.057, Fig. 3G and H). By stratifying ACC patients for dichotomized Ki67 LI (cut-off value = 20%), the Kaplan–Meier curves revealed weak prognostic power (OS, log-rank = 0.031; DFS, log-rank = 0.088). Significant differences in time to survival (TtS) and TtR were recorded only when grouping patients for the presence or absence of necrosis (34 [12–78] vs 57.5 [31.5–103.5] months, *p* = 0.05 for TtS and 7 [1.0–31.5] vs 57.5 [31.5–103.5] months, *p* < 0.001 for TtR). No significant differences in the OS and DFS curves were observed for the other Weiss parameters (data not shown). When the Kaplan–Meier analysis was performed in R0 patients, necrosis remained significant for both OS and DFS (Fig. 3I and J), while all other variables (stage, size, venous invasion) lost their predictive value (data not shown).

Finally, we estimated the risk of recurrence and death by multivariate Cox regression analysis (Table 6). Necrosis, considered separately or combined with venous invasion as Component 1, retained significance in predicting risk of death and recurrence after adjustment for age, tumor size and total Weiss score.

**Table 6:** Multivariate Cox regression analyses of hazard ratio for ACC recurrence and death

Variable	Recurrence			Death		
	HR	95% CI	*p*	HR	95% CI	*p*
Necrosis	12.2	[1.6–95.0]	*0.017*	7.8	[1.0–63.5]	*0.050*
Venous invasion	0.6	[0.6–2.16]	0.588	1.0	[0.4–2.5]	0.974
Age	1.0	[1.0–1.1]	*0.011*	1.0	[1.0–1.1]	*0.014*
Tumor size	1.0	[1.0–1.1]	0.149	1.0	[0.9–1.1]	0.615
Total Weiss score	1.1	[0.8–1.4]	0.549	1.1	[0.8–1.5]	0.551

	**Recurrence**			**Death**		
	HR	95% CI	*p*	HR	95% CI	*p*
Component 1	1.8	[1.1–3.0]	*0.016*	2.1	[1.1–4.1]	*0.022*
Age	1.1	[1.0–1.1]	*0.004*	1.0	[1.0–1.1]	*0.003*
Tumor size	1.1	[1.0–1.1]	*0.010*	1.0	[1.0–1.1]	0.507
Total Weiss score	1.3	[1.0–1.7]	*0.014*	1.3	[1.0–1.7]	*0.050*

Hazard ratio (HR) and 95% confidence intervals (CI) are indicated for multivariate analysis; *p* values are indicated (significant in italics)

## Discussion

The high mortality and limited therapeutic options currently available for ACC require accurate patient stratification for deciding on personalized and precise treatment [14–16]. In a two ENSAT center-based ACC cohort of 79 patients, we applied PCA to Weiss parameters to reduce the number of non-correlated variables and evaluate their relative weight. We also analyzed the prognostic value of individual Weiss score criteria in combination with more traditional variables (e.g., tumor size, TNM stage, margin status). Only necrosis and its relevant PCA component proved to significantly predict patient survival, also retaining its prognostic ability in the R0 subgroup.

To the best of our knowledge, this is the first application of an un-hierarchical PCA approach to simplify and prioritize Weiss score parameters. Coste et al. previously used Rasch model analysis to assess the unidimensionality of the Weiss score, concluding that the biases generated by differential item functioning (DIF) due to the size and weight of the tumor for two items (diffuse architecture and necrosis) may be substantial [17]. However, the complementary factor analyses they conducted was not un-hierarchical and was run on three pre-fixed domains (nucleus, architecture, and invasion), thus forcing specific association between parameters [17].

In our study, PCA was unrestrained and grouped the nine parameters into four rather than three independent components, with each parameter unequivocally related to one specific component. Differently from Coste et al., necrosis did not cluster in the architecture domain, but combined with venous invasion in Component 1. We can speculate that this finding may be relevant to tumor metastatic potential. Intratumoral hypoxia, histologically reflected by the presence of necrosis, can lead to the development of an aggressive phenotype, which includes cell immortalization and dedifferentiation, pH regulation, autocrine growth/survival, angiogenesis, and invasion/metastasis.

Lymphatic (sinusoidal) and capsular invasion clustered with diffuse tumor architecture in Component 2, while Component 3 included the cytologic items (high nuclear grade, high mitotic rate, and atypical mitoses). Finally, Component 4 comprised the low prevalence of lipid-rich or clear cells in the tumor mass (≤ 25%), a marker of adrenal cortical differentiation. Cell lipid content and quality have recently been suggested to be highly relevant for shaping cancer cell phenotype [18], and Sterol O-Acyl Transferase 1 (SOAT1), a key enzyme for cholesterol esterification, has been established as an independent predictor of death and recurrence in ACC patients [19]. The reciprocal crosstalk lately demonstrated between adipose-derived stem cells and the ACC cell line H295R confirms the relevance of lipid metabolism in cancer cell reprogramming and invasion [20, 21]

Interestingly, Component 1 was not only the main measure of Weiss distribution variability at PCA, but also the only component capable of significantly predicting OS and DFS at the Kaplan–Meier analysis. In our series, necrosis was not detected in all tumors, being present in 70% of low stages (I–II) and absent in 11% of high stages (III–IV). This finding further underlines the prognostic value of histologic coagulative tumor necrosis, comparable to conventional parameters such as tumor stage (I–II vs III–IV) and size (cut-off 6 cm).

Ki67 proliferation index has been suggested to be a strong prognostic indicator in both localized and advanced ACC [1, 22]. However, the Ki67 scoring assessment may differ considerably, and both inter- and intra-observer variability poses limitations to its clinical utility. In our study, Ki67 index revealed poor predictive power (DFS: log-rank = 0.088; OS: log-rank = 0.031 for cut-off 20%), probably due to the high proportion of missing values. Despite our efforts to revise the Ki67 LI for all patients, we were able to retrieve only 26 (33%) of 79 tumor samples for immunohistochemical examination and centralized revision.

We acknowledge some limitations of our study: (i) the retrospective nature of the study and long recruitment period, which might have affected variance in some analyses; (ii) incomplete dataset for Ki67 scoring assessment, with only one-third of values available; (iii) the absence of a validation cohort. The main strengths of the study are (i) the relatively large cohort of patients from two independent ENSAT centers, (ii) the good quality of our data, and (iii) the un-hierarchical PCA approach employed.

In conclusion, PCA applied to the Weiss scoring system suggests that necrosis is the most powerful adverse factor and the best histologic predictor of OS and DFS in ACC. Future prospective trials with centralized pathologic evaluation should be conducted to validate these preliminary results.

## Data Availability

The datasets generated during the current study will be available upon reasonable request.

## References

[1] Libé R, Borget I, Ronchi CL, Zaggia B, Kroiss M, Kerkhofs T, Bertherat J, Volante M, Quinkler M, Chabre O, Bala M, Tabarin A, Beuschlein F, Vezzosi D, Deutschbein T, Borson-Chazot F, Hermsen I, Stell A, Fottner C, Leboulleux S, Hahner S, Mannelli M, Berruti A, Haak H, Terzolo M, Fassnacht M, Baudin E, ENSAT network,  (2015). Prognostic factors in stage III-IV adrenocortical carcinomas (ACC): an European Network for the Study of Adrenal Tumor (ENSAT) study. Ann Oncol.

[2] Armignacco R, Cantini G, Canu L, Poli G, Ercolino T, Mannelli M, Luconi M (2018). Adrenocortical carcinoma: the dawn of a new era of genomic and molecular biology analysis. J Endocrinol Invest.

[3] Assié G, Jouinot A, Fassnacht M, Libé R, Garinet S, Jacob L, Hamzaoui N, Neou M, Sakat J, de La Villéon B, Perlemoine K, Ragazzon B, Sibony M, Tissier F, Gaujoux S, Dousset B, Sbiera S, Ronchi CL, Kroiss M, Korpershoek E, de Krijger R, Waldmann J, Quinkler M, Haissaguerre M, Tabarin A, Chabre O, Luconi M, Mannelli M, Groussin L, Bertagna X, Baudin E, Amar L, Coste J, Beuschlein F, Bertherat J (2019). Value of molecular classification for prognostic assessment of adrenocortical carcinoma. JAMA Oncol.

[4] Clay MR, Pinto EM, Fishbein L, Else T, Kiseljak-Vassiliades K (2022). Pathological and genetic stratification for management of adrenocortical carcinoma. J Clin Endocrinol Metab.

[5] Mete O, Erickson LA, Juhlin CC, de Krijger RR, Sasano H, Volante M, Papotti MG (2022). Overview of the 2022 WHO classification of adrenal cortical tumors. Endocr Pathol.

[6] Lalli E, Luconi M (2018). The next step: mechanisms driving adrenocortical carcinoma metastasis. Endocr Relat Cancer.

[7] Cantini G, Canu L, Armignacco R, Salvianti F, De Filpo G, Ercolino T, Nesi G, Maggi M, Mannelli M, Pinzani P, Luconi M (2020). Prognostic and monitoring value of circulating tumor cells in adrenocortical carcinoma: a preliminary monocentric study. Cancers (Basel).

[8] WHO Classification of Tumours Editorial Board (2022) WHO classification of endocrine and neuroendocrine tumours. IARC, France, Lyon

[9] Pennanen M, Heiskanen I, Sane T, Remes S, Mustonen H, Haglund C, Arola J (2015). Helsinki score-a novel model for prediction of metastases in adrenocortical carcinomas. Hum Pathol.

[10] Duregon E, Fassina A, Volante M, Nesi G, Santi R, Gatti G, Cappellesso R, Dalino Ciaramella P, Ventura L, Gambacorta M, Dei Tos AP, Loli P, Mannelli M, Mantero F, Berruti A, Terzolo M, Papotti M (2013). The reticulin algorithm for adrenocortical tumor diagnosis: a multicentric validation study on 245 unpublished cases. Am J Surg Pathol.

[11] Jolliffe IT (2002) Principal Component Analysis. Springer Series in Statistics. Springer, New York. 10.1007/0-387-22440-8_13

[12] Lau SK, Weiss LM (2009). The Weiss system for evaluating adrenocortical neoplasms: 25 years later. Hum Pathol.

[13] Fassnacht M, Johanssen S, Quinkler M, Bucsky P, Willenberg HS, Beuschlein F, Terzolo M, Mueller HH, Hahner S, Allolio B; German Adrenocortical Carcinoma Registry Group; European Network for the Study of Adrenal Tumors (2009). Limited prognostic value of the 2004 International Union Against Cancer staging classification for adrenocortical carcinoma: proposal for a Revised TNM Classification. Cancer.

[14] Duregon E, Cappellesso R, Maffeis V, Zaggia B, Ventura L, Berruti A, Terzolo M, Fassina A, Volante M, Papotti M (2017). Validation of the prognostic role of the "Helsinki Score" in 225 cases of adrenocortical carcinoma. Hum Pathol.

[15] Baechle JJ, Marincola Smith P, Solórzano CC, Tran TB, Postlewait LM, Maithel SK, Prescott J, Pawlik T, Wang TS, Glenn J, Hatzaras I, Shenoy R, Phay JE, Shirley LA, Fields RC, Jin L, Abbott DE, Ronnekleiv-Kelly S, Sicklick JK, Yopp A, Mansour J, Duh QY, Seiser N, Votanopoulos K, Levine EA, Poultsides G, Kiernan CM (2021). Cumulative GRAS score as a predictor of survival after resection for adrenocortical carcinoma: analysis from the U.S. adrenocortical carcinoma database. Ann Surg Oncol.

[16] Giordano TJ, Berney D, de Krijger RR, Erickson L, Fassnacht M, Mete O, Papathomas T, Papotti M, Sasano H, Thompson LDR, Volante M, Gill AJ (2021). Data set for reporting of carcinoma of the adrenal cortex: explanations and recommendations of the guidelines from the International Collaboration on Cancer Reporting. Hum Pathol.

[17] Coste J, Tissier F, Pouchot J, Ecosse E, Rouquette A, Bertagna X, Libé R, Viallon V (2014). Rasch analysis for assessing unidimensionality and identifying measurement biases of malignancy scores in oncology. The example of the Weiss histopathological system for the diagnosis of adrenocortical cancer. Cancer Epidemiol.

[18] Warde KM, Lim YJ, Ribes Martinez E, Beuschlein F, O'Shea P, Hantel C, Dennedy MC (2022) Mitotane targets lipid droplets to induce lipolysis in adrenocortical carcinoma. Endocrinology 163: bqac102. 10.1210/endocr/bqac102 10.1210/endocr/bqac102 PMC9342684 35797592

[19] Lacombe AMF, Soares IC, Mariani BMP, Nishi MY, Bezerra-Neto JE, Charchar HDS, Brondani VB, Tanno F, Srougi V, Chambo JL, Costa de Freitas RM, Mendonca BB, Hoff AO, Almeida MQ, Weigand I, Kroiss M, Zerbini MCN, Fragoso MCBV (2020). Sterol O-acyl transferase 1 as a prognostic marker of adrenocortical carcinoma. Cancers (Basel).

[20] Armignacco R, Cantini G, Poli G, Guasti D, Nesi G, Romagnoli P, Mannelli M, Luconi M (2019). The adipose stem cell as a novel metabolic actor in adrenocortical carcinoma progression: evidence from an in vitro tumor microenvironment crosstalk model. Cancers (Basel).

[21] Cantini G, Di Franco A, Mannelli M, Scimè A, Maggi M, Luconi M (2020). The role of metabolic changes in shaping the fate of cancer-associated adipose stem cells. Front Cell Dev Biol.

[22] Beuschlein F, Weigel J, Saeger W, Kroiss M, Wild V, Daffara F, Libé R, Ardito A, Al Ghuzlan A, Quinkler M, Oßwald A, Ronchi CL, de Krijger R, Feelders RA, Waldmann J, Willenberg HS, Deutschbein T, Stell A, Reincke M, Papotti M, Baudin E, Tissier F, Haak HR, Loli P, Terzolo M, Allolio B, Müller HH, Fassnacht M (2015). Major prognostic role of Ki67 in localized adrenocortical carcinoma after complete resection. J Clin Endocrinol Metab.

